# 14‐3‐3‐*β* and ‐*ε* contribute to activation of the osmoprotective transcription factor NFAT5 by increasing its protein abundance and its transactivating activity

**DOI:** 10.14814/phy2.12000

**Published:** 2014-04-23

**Authors:** Yuichiro Izumi, Maurice B. Burg, Joan D. Ferraris

**Affiliations:** 1 Systems Biology Center, National Heart Lung and Blood Institute, National Institutes of Health, Bethesda, Maryland

**Keywords:** 14‐3‐3, NFAT5, osmotic, protein stability

## Abstract

Having previously found that high NaCl causes rapid exit of 14‐3‐3 isoforms from the nucleus, we used siRNA‐mediated knockdown to test whether 14‐3‐3s contribute to the high NaCl‐induced increase in the activity of the osmoprotective transcription factor NFAT5. We find that, when NaCl is elevated, knockdown of 14‐3‐3‐*β* and/or 14‐3‐3‐*ε* decreases NFAT5 transcriptional activity, as assayed both by luciferase reporter and by the mRNA abundance of the NFAT5 target genes aldose reductase and the sodium‐ and chloride‐dependent betaine transporter, BGT1. Knockdown of other 14‐3‐3 isoforms does not significantly affect NFAT5 activity. 14‐3‐3‐*β* and/or 14‐3‐3‐*ε* do not act by affecting the nuclear localization of NFAT5, but by at least two other mechanisms: (1) 14‐3‐3‐*β* and 14‐3‐3‐*ε* increase protein abundance of NFAT5 and (2) they increase NFAT5 transactivating activity. When NaCl is elevated, knockdown of 14‐3‐3‐*β* and/or 14‐3‐3‐*ε* reduces the protein abundance of NFAT5, as measured by Western blot, without affecting the level of NFAT5 mRNA, and the knockdown also decreases NFAT5 transactivating activity, as measured by luciferase reporter. The 14‐3‐3s increase NFAT5 protein, not by increasing its translation, but by decreasing the rate at which it is degraded, as measured by cycloheximide chase. It is not clear at this point whether the 14‐3‐3s affect NFAT5 directly or indirectly through their effects on other proteins that signal activation of NFAT5.

## Abstract (precis)

e12000

When NaCl is elevated, knockdown of 14‐3‐3‐*β* and/or 14‐3‐3‐*ε* reduces the protein abundance of NFAT5, as measured by Western blot, without affecting the level of NFAT5 mRNA, and the knockdown also decreases NFAT5 transactivating activity, as measured by luciferase reporter. The 14‐3‐3s increase NFAT5 protein, not by increasing its translation, but by decreasing the rate at which it is degraded, as measured by cycloheximide chase.

## Introduction

14‐3‐3 proteins (Tzivion and Avruch 2002; Bridges and Moorhead 2005; Aitken 2006) modulate numerous cellular processes, including responses to environmental stress. They are small (~30 kD), abundant, acidic proteins. Mammals express seven highly conserved isoforms (*β*,* ε*,* η*,* γ*,* τ*,* ζ*, and *σ*) that have high amino acid sequence similarity. 14‐3‐3s bind to phosphopeptides in hundreds of different target proteins. Two phosphopeptide motifs in the targets (RSXpSXP and RXXXpSXP) bind most 14‐3‐3 isoforms. Nevertheless, target proteins often do not contain sequences that conform precisely to these motifs, and in a few instances 14‐3‐3 proteins have been observed to bind unphosphorylated targets. The 14‐3‐3 isoforms form both homo‐ and heterodimers. Various 14‐3‐3 isoforms have similar binding specificities, as would be anticipated from their structure, since the residues lining their phosphopeptide‐binding grooves are highly conserved. Nevertheless, in many cases, the interacting proteins show a distinct preference for particular isoforms of 14‐3‐3. The isoforms presumably have distinct functions, but knockouts of a number of 14‐3‐3 isoforms did not produce definite phenotypes, possibly reflecting the fact that isoforms may replace each other. Binding of a 14‐3‐3 dimer to its target can have a variety of effects. The binding can alter the ability of the target protein to interact with other partners; modify the cytoplasmic/nuclear partition of the target protein by increasing its nuclear export rate, decreasing its nuclear import, or both; inhibit or augment the intrinsic catalytic activity of the target protein; and protect the target protein from proteolysis and/or dephosphorylation. Ligand‐free 14‐3‐3 proteins form highly helical, cup‐shaped dimers. This highly rigid structure leads to deformation of the target protein with little or no change in the structure of the 14‐3‐3 dimer. Also, each subunit of the dimer is able to bind a discrete phosphoserine‐ or phosphothreonine‐containing ligand independently, which brings the ligands into juxtaposition. The phosphorylation of a 14‐3‐3 isoform on specific residues can regulate its function.

NFAT5 (Miyakawa et al. 1999; Ko et al. 2000; Burg et al. 2007) (also called TonEBP or OREBP) was originally identified as a transcription factor that increases RNA and protein abundance of genes that protect cells from adverse effects of hypertonicity, including the hypertonicity produced by high extracellular NaCl in the kidney medulla. Subsequently, numerous additional roles of NFAT5 have been identified, some of which apparently do not involve hypertonicity (Halterman et al. 2012). Hypertonicity increases NFAT5 activity by several complementary effects. High NaCl increases mRNA and protein abundance of NFAT5, itself (Miyakawa et al. 1999; Ko et al. 2000), causes NFAT5 to move from the cytoplasm into the nucleus (Miyakawa et al. 1999; Ko et al. 2000) and increases transactivating activity of NFAT5 (Ferraris et al., 2002b). Hypertonicity alters activity of numerous kinases and phosphatases, resulting in altered phosphorylation of amino acids in NFAT5 that regulates its activity.

The possibility that 14‐3‐3s might contribute to osmoregulation of NFAT5 occurred to us following a proteomic screen for proteins whose nuclear/cytoplasmic distribution is affected by high NaCl (Li et al. 2012). We found that high NaCl causes rapid exit of 14‐3‐3 isoforms from the nucleus. The purpose of the present experiments was to test whether 14‐3‐3s contribute to osmotic regulation of NFAT5, and, if so, which 14‐3‐3 isoforms are involved and by what mechanism(s). We tested the effects of 14‐3‐3 isoforms by using siRNAs to knock them down in the presence or absence of high NaCl.

## Materials and Methods

### Reagents, antibodies, and siRNAs

ON‐TARGETplus SMARTpool siRNAs against 14‐3‐3 isoforms and scramble control siRNAs were from Thermo Scientific (Pittsburgh, PA), Lipofectamine 2000 from Invitrogen Life Technologies (Grand Island, NY), cycloheximide from Sigma‐Aldrich (St. Louis, MO). Rabbit anti‐NFAT5 antibody (sc‐13035), rabbit and mouse anti‐14‐3‐3*β* antibodies (sc‐628 and sc‐594), rabbit anti‐14‐3‐3*γ* antibody (sc‐731), goat anti‐14‐3‐3*η* antibody (sc‐17286), rabbit anti‐14‐3‐3*θ* antibody (sc‐732), rabbit anti‐14‐3‐3*ζ* antibody (sc‐1019), rabbit anti‐HSP90*α*/*β* antibody (sc7947), and rabbit anti‐CDK5 antibody (sc‐173) were purchased from Santa Cruz Biotechnology (Santa Cruz, CA). Mouse anti‐*α* tubulin antibody (691251) was purchased from MP Biomedicals (Solon, OH). Rabbit anti‐CREB antibody (9197S) was purchased from Cell signaling (Danvers, MA). Mouse anti‐V5 antibody (MCA1360) was purchased from AbD Serotec (Raleigh, NC). Luciferase Assay System was from Promega (Madison, WI), NE‐PER nuclear and cytoplasmic extraction reagent kit from Thermo Scientific, and PhosphoSafe Extraction Reagent from EMD Millipore (Gibbstown, NJ).

### Cell culture and treatment

Human embryonic kidney 293 (HEK293) cells were purchased from ATCC (Manassas, VA) and grown in EMEM plus 10% FBS in 5% CO_2_/95% air at 37°C. All experiments were performed with 3–6 bioreplicates using HEK293 passages 42–51. HEK293 cells stably expressing ORE‐X‐ or TAD‐luciferase reporters were established as previously described (Irarrazabal et al. 2004). For knock down of 14‐3‐3s, cells were seeded on six‐well plates and reverse transfected with siRNAs at 5 or 10 nmol/L, using Lipofectamine 2000. Three days after transfection, the medium was changed to an otherwise identical one at 300 or 500 mosmol/kg (NaCl added) for the indicated time. Cells were harvested with PhosphoSafe Extraction Reagent for Western blot or Reporter Lysis Buffer for Luciferase reporter assay. siRNAs were used at 5 and 10 nmol/L. Cycloheximide was dissolved in DMSO and used at 100 µg/mL. Luciferase activity, measuring transcriptional or transactivating activities, was assayed using a Victor3 (PerkinElmer, Waltham, MA) and normalized to total protein.

### Western analysis

Total cell protein was extracted using PhosphoSafe Extraction Reagent. Nuclear and cytoplasmic proteins were extracted using the NE‐PER nuclear and cytoplasmic extraction reagent kit (Pierce, Rockford, IL), according to the manufacturer's instructions. The total concentration of extracted proteins was measured using BCA protein assay reagent (Thermo Scientific). Twenty micrograms of total protein were run on a NuPAGE Novex 3–8% tris‐acetate gel or 4–12% Bis‐Tris gel (Invitrogen) and transferred to a nitrocellulose membrane (Invitrogen) by electrophoresis. The membrane was incubated in Odyssey blocking buffer (LI‐COR Biosciences, Lincoln, NE) for 1 h, then incubated with primary antibody overnight, followed by IR dye‐labeled secondary antibody for 1 h. The membrane was scanned using the Odyssey imaging system (LI‐COR BioSciences). The nuclear to cytoplasmic ratio was calculated as previously described (Ferraris and Burg 2007). Tubulin was used as a loading control. CREB and tubulin were used as nuclear and cytoplasmic markers, respectively.

### Immunoprecipitation

Ninety percent confluent HEK293 cells were transfected with pcDNA6‐empty‐V5 or pcDNA‐NFAT5‐V5 (NFAT5, isoform c, NP_006590) constructs using Lipofectamine 2000. After 24 h, cells were incubated at 300 or 500 mosmol/kg (NaCl varied) for 1 h and then harvested with PhosphoSafe Extraction Reagent. Cell extracts were preincubated (preclearing step) with Dynabeads (Invitrogen) at 4°C for 1 h. Precleared cell extracts were incubated with anti‐V5 antibody conjugated Dynabeads (Invitrogen).

### Real‐time PCR

Messenger RNA abundance was measured by real‐time PCR using TaqMan gene expression assay. Total RNAs were extracted by RNeasy Mini Kit (Qiagen, Valencia, CA). One hundred nanograms total RNA was reverse transcribed to cDNA, and primer‐amplified using High Capacity cDNA Reverse Transcription Kit (Applied Biosystems, Life Technologies, Austin, TX). mRNA abundance relative to control (such as scramble siRNA transfection at 300 mosmol) was calculated as previously published (Cai et al. 2004). Primer sets for NFAT5 (Hs00232437_m1) and 18s rRNA (4310893E) were from Applied Biosystems, Life Technologies. Primer sets for AR (Ferraris et al., 2002a) and BGT1 (Ferraris et al., 2002b) were as previously designed. We confirmed equal abundances of 18s rRNA among samples (data not shown). Target gene mRNA was not normalilized to 18s rRNA.

### Statistics

**Figure 1.**
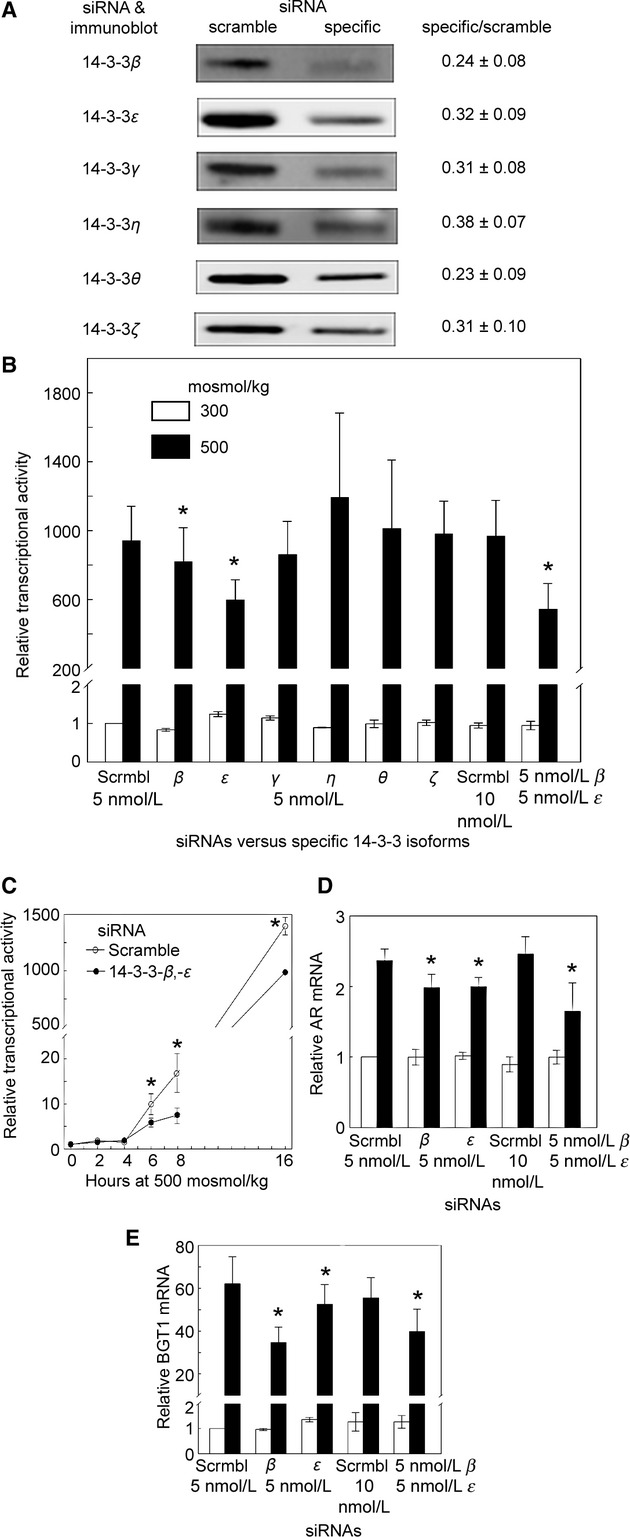
(A) Effectiveness of siRNAs versus 14‐3‐3s. HEK293 cells were transfected with 5 nmol/L siRNAs for 3 days, then cell proteins were extracted, and abundance of 14‐3‐3 proteins measured by Western analysis. siRNAs knock 14‐3‐3s down by 62–77%. Center panel shows representative Western blots. Right panel shows mean ± SEM,* n* = 3. (B) Effect of knock down of 14‐3‐3s on transcriptional activity of NFAT5. HEK293 cells that stably express the ORE‐X luciferase reporter of NFAT5 transcriptional activity were used. The siRNAs were transfected for 3 days into cells incubated at 300 mosmol/kg, then the medium was changed, keeping the osmolality at 300 or increasing it to 500 mosmol/kg for 16 h by adding NaCl. None of the siRNAs significantly affect the transcriptional activity at 300 mosmol/kg, but at 500 mosmol/kg siRNAs against 14‐3‐3‐*β* or ‐*ε* inhibit NFAT5 transcriptional activity by 15% and 35%, respectively, and knock down of ‐*β* and ‐*ε* in combination inhibits ORE‐X activity by 45% (mean ± SEM, *P < 0.05, *n* = 3–6). (C) Time course of the high NaCl‐induced increase in NFAT5 transcriptional activity. As in (B), except that NFAT5 transcriptional activity was measured at the times shown. Effect of knock down of 14‐3‐3‐*β* and ‐*ε* on mRNA expression of the NFAT5 target genes aldose reductase (AR) (D) and BGT1 (E). As in (B), except that AR or BGT1 mRNA was measured in HEK293 cells (mean ± SEM, *P < 0.05, *n* = 3).

Results are displayed as mean ± SEM. Statistical significance was determined by paired *t*‐test (Fig. 1A) or ANOVA and Bonferroni multiple comparison (all other data). *P *<**0.05 was considered significant.

## Results

### Effect on transcriptional activity of NFAT5 of knocking down 14‐3‐3 isoforms individually and in combination

We measured the contribution of 14‐3‐3s to NFAT5 transcriptional activity by siRNA‐mediated knock down of the 14‐3‐3s in HEK293 cells that stably express a luciferase reporter of NFAT5 transcriptional activity (ORE‐X) (Irarrazabal et al. 2004). The siRNAs knock down expression of various 14‐3‐3s by 62–77% within 3 days (Fig. 1A). HEK293 cells that stably express the ORE‐X luciferase reporter of NFAT5 transcriptional activity were used to measure the effect of the siRNAs on NFAT5 transcriptional activity (Fig. 1B). The siRNAs were transfected for 3 days into cells incubated at 300 mosmol/kg, then the medium was changed, keeping the osmolality at 300 or increasing it to 500 mosmol/kg for 16 h by adding NaCl. None of the siRNAs significantly affect the transcriptional activity at 300 mosmol/kg, but at 500 mosmol/kg siRNAs against 14‐3‐3‐*β* or ‐*ε* inhibit NFAT5 transcriptional activity by 15% and 35%, respectively, and knock down of ‐*β* and ‐*ε* in combination inhibits ORE‐X activity by 45%. When we examined shorter exposures to hypertonicity, knock down of 14‐3‐3‐*β* and ‐*ε* in combination have a maximum effect on ORE‐X activity at 8 h (50% reduction at 500 mosmol/kg) (Fig. 1C).

### Effect on mRNA abundance of the NFAT5 target genes aldose reductase and BGT1 of knocking down 14‐3‐3‐*β* and ‐*ε*

In order further to test whether 14‐3‐3‐*β* and ‐*ε* contribute to regulation of NFAT5 activity, we examined the effect of knocking them down on mRNA expression of the NFAT5 target genes aldose reductase (AR) (Ferraris et al. 1994) and the sodium‐ and chloride‐dependent betaine transporter (BGT1) (Takenaka et al. 1994). At 300 mosmol/kg knocking down 14‐3‐3‐*β* and/or 14‐3‐3‐*ε* does not affect aldose reductase (AR) mRNA abundance (Fig. 1D). However, at 500 mosmol/kg knock down of either 14‐3‐3‐*β* or 14‐3‐3‐*ε* decreases aldose reductase (AR) mRNA abundance by 15%, and knock down of *β* and *ε* in combination decreases AR mRNA abundance by 30% (Fig. 1D). The results with BGT1 mRNA are similar. At 300 mosmol/kg knocking down 14‐3‐3‐*β* and/or 14‐3‐3‐*ε* does not affect BGT1 mRNA abundance (Fig. 1E). However, at 500 mosmol/kg knock down of 14‐3‐3‐*β* and ‐*ε* individually decreases BGT1 mRNA abundance by 45% and 15%, respectively, and knock down of ‐*β* and ‐*ε* in combination decreases BGT1 mRNA abundance by 30% (Fig. 1E). Having found that 14‐3‐3‐*β* and ‐*ε* contribute to the high NaCl‐induced increase in NFAT5 transcriptional activity, we turned our attention to identifying the mechanism involved. High NaCl increases NFAT5 activity by a combination of effects: greater NFAT5 abundance (Miyakawa et al. 1999; Ko et al. 2000), increased nuclear localization of NFAT5 (Miyakawa et al. 1999; Ko et al. 2000), and elevated NFAT5 transactivating activity (Ferraris et al., 2002b). Our next experiments were aimed at distinguishing which of these are regulated by 14‐3‐3‐*β* and ‐*ε*.

### Effect on transactivating activity of NFAT5 of knock down of 14‐3‐3 isoforms individually or in combination

**Figure 2.**
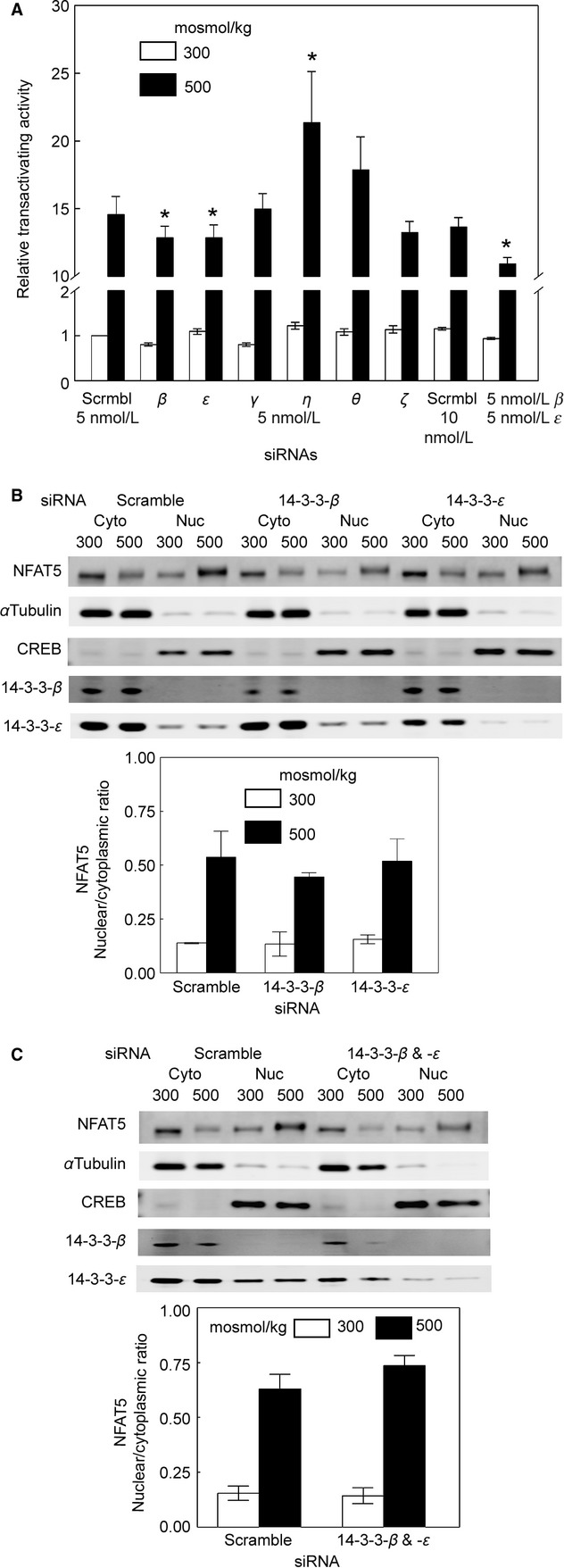
(A) Effect of knock down of 14‐3‐3s on transactivating activity of NFAT5. As in (Fig. 1B), except that the luciferase reporter stably expressed in HEK293 cells measures NFAT5 transactivating activity. The siRNAs do not significantly affect the transactivating activity at 300 mosmol/kg siRNA, but at 500 mosmol/kg siRNA against 14‐3‐3‐*β* or ‐*ε* inhibits transactivating activity by 10%, and knock down of ‐*β* and ‐*ε* in combination inhibits transactivating activity by 20% (mean ± SEM, *P <**0.05, *n *=**3–7). (B) Effect of knock down of 14‐3‐3‐*β* or ‐*ε* on nuclear/cytoplasmic ratio of NFAT5; 5 nmol/L siRNAs against 14‐3‐3‐*β* or ‐*ε* was transfected for 3 days into cells incubated at 300 mosmol/kg, then the medium was changed, keeping the osmolality at 300 or increasing it to 500 mosmol/kg for 1 h by adding NaCl. High NaCl‐induced nuclear localization of NFAT5 is largely complete within 1 h. Nuclear and cytoplasmic proteins were extracted separately and quantified by Western blot. The siRNAs do not significantly change the nuclear to cytoplasmic ratio of NFAT5 (*P <**0.05, *n *=**3). (C) Effect of knock down of 14‐3‐3‐*β* and ‐*ε* in combination on nuclear/cytoplasmic ratio of NFAT5. As in (B) except that the siRNAs were combined. The siRNAs in combination do not significantly change the nuclear to cytoplasmic ratio of NFAT5 (*P <**0.05, *n *=**3).

We used HEK293 cells that stably express a binary luciferase reporter of NFAT5 transactivating activity (Ferraris et al., 2002b). Knock down of the 14‐3‐3s does not affect transactivating activity of NFAT5 at 300 mosmol/kg (Fig. 2A). However, at 500 mosmol/kg siRNA against 14‐3‐3‐*β* or ‐*ε* inhibits ORE‐X activity by 10%, and knock down of ‐*β* and ‐*ε* in combination inhibits transactivating activity by 20% (Fig. 2A). Since the effect on transactivating activity is considerably less than the effect on transcriptional activity, we searched for additional effects of 14‐3‐3‐*β* and ‐*ε* that might contribute to their regulation of NFAT5 transcriptional activity. Note in addition, that siRNA against 14‐3‐3‐*η* significantly increases NFAT5 transactivating activity at 500 mosmol/kg (Fig. 2A). However, since 14‐3‐3‐*η* does not increase NFAT5 transcriptional activity to a statistically significant extent (Fig. 1B), we chose not to investigate 14‐3‐3‐*η* further.

### Lack of effect on nuclear/cytoplasmic ratio of NFAT5 of knock down of 14‐3‐3‐*β* and ‐*ε*

Knock down of 14‐3‐3‐*β* and ‐*ε*, whether individually (Fig. 2B) or in combination (Fig. 2C) does not significantly change the nuclear to cytoplasmic ratio of NFAT5. siRNAs against 14‐3‐3‐*β* and ‐*ε* do not cross‐react with each other either at the protein level (Fig. 2B) or at the mRNA level (data not shown). We conclude that 14‐3‐3‐*β* and ‐*ε* do not regulate NFAT5 activity by altering its nuclear translocation.

### Effect on NFAT5 protein abundance of knock down of 14‐3‐3‐*β* and ‐*ε*

**Figure 3.**
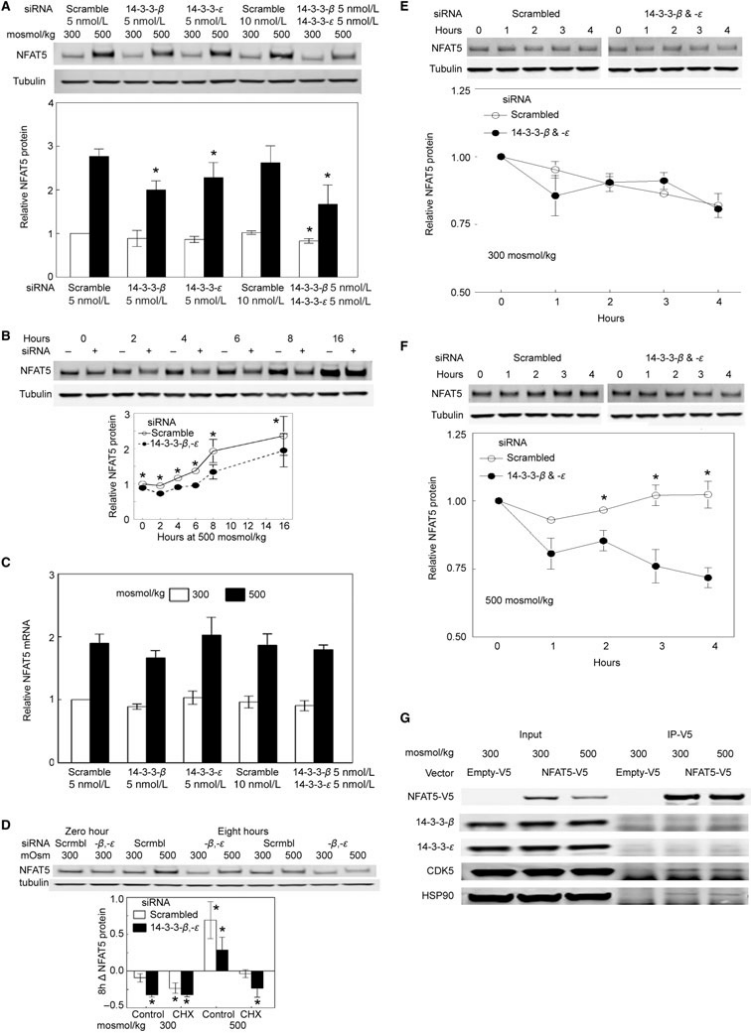
Effect of knock down of 14‐3‐3‐*β* and/or ‐*ε* on protein abundance of NFAT5. (A) Knock down of 14‐3‐3‐*β* and/or ‐*ε* reduces NFAT5 protein abundance. siRNAs against 14‐3‐3‐*β* and/or ‐*ε* were transfected for 3 days into HEK293 cells incubated at 300 mosmol/kg, then the medium was changed, keeping the osmolality at 300 or increasing it to 500 mosmol/kg for 8 h by adding NaCl. Upper panel is a representative Western blot. The siRNAs singly and in combination reduce NFAT5 protein abundance (mean ± SEM, *n *=**3, *P <**0.05). (B) Time course of the effect of 14‐3‐3‐*β* and ‐*ε* in combination. As in (A) except that NFAT5 protein was measured just before NaCl was increased (zero time) and after that at the intervals that are shown. Upper panel is a representative Western blot (Mean ± SEM, *P <**0.05, *n *=**3). (C) Lack of effect of 14‐3‐3‐*β* and/or ‐*ε* separately or combined on NFAT5 mRNA abundance. As in (A) except that NFAT5 mRNA was measured 16 h after NaCl was increased (*P *>**0.05, *n *=**3). (D–F) siRNA‐mediated knockdown of 14‐3‐3‐*β* and ‐*ε* in combination increases degradation of NFAT5 protein. siRNAs against 14‐3‐3‐*β* and ‐*ε* were transfected for 3 days into HEK293 cells incubated at 300 mosmol/kg. (D) One hundred μg/mL cycloheximide (CHX) or vehicle (DMSO control) was added for 1 h before changing to otherwise identical media kept at 300 mosmol/kg or increased to 500 mosmol/kg by adding NaCl. Media contained CHX or DMSO. NFAT5 protein was measured by Western blot at the start and end of the 8 h increase in NaCl, and the difference (Δ) was calculated. (*P <**0.05, *n *=**3). (E and F) Measurement of the rate of NFAT5 degradation by cycloheximide chase. HEK293 cells were preincubated for 7 h at 300 or 500 mosmol/kg, then 100 μg/mL cycloheximide was added for 1 h. Cells maintained in cycloheximide were harvested at the intervals shown. (*P <**0.05, *n *=**3–4). (G) Lack of coimmunoprecipitation of 14‐3‐3‐*β* and ‐*ε* with NFAT5. NFAT5‐V5 was immunoprecipitated from HEK293 cells, then the immunoprecipitates were immunoblotted for the proteins shown. CDK5 and HSP90 coimmunoprecipitate with NFAT5‐V5, but 14‐3‐3‐*β* and ‐*ε* do not.

High NaCl increases the protein abundance of NFAT5 (Miyakawa et al. 1999; Woo et al. 2000). If osmolality is left at 300 mosmol/kg, siRNA‐mediated knock down of 14‐3‐3‐*β* and ‐*ε*, separately does not significantly affect the protein abundance of NFAT5 in HEK293 cells, but, knocking them down in combination reduces NFAT5 protein by 20% (Fig. 3A). When osmolality is increased to 500 mosmol/kg by adding NaCl for 8 h, siRNA against 14‐3‐3‐*β* reduces NFAT5 protein abundance by 30%, siRNA against 14‐3‐3‐*ε* reduces NFAT5 protein abundance by 20%, and the siRNAs in combination reduce NFAT5 protein abundance by 40% (Fig. 3A). Furthermore, siRNAs against 14‐3‐3‐*β* and ‐*ε* in combination delay the high NaCl‐induced increase in NFAT5 protein and reduce the increase for at least 16 h (Fig. 3B).

### Effect of knock down of 14‐3‐3‐*β* and ‐*ε* on NFAT5 protein stability

High NaCl increases NFAT5 mRNA, resulting in increased NFAT5 translation and protein abundance (Miyakawa et al. 1999; Ko et al. 2000; Woo et al. 2000; Cai et al. 2005). However, siRNAs against 14‐3‐3‐*β* and 14‐3‐3‐*ε* separately and combined do not affect NFAT5 mRNA abundance at 300 or 500 mosmol/kg (Fig. 3C). Therefore, we turned to the possibility that that the 14‐3‐3s increase NFAT5 protein by reducing the rate at which it is degraded. To study NFAT5 degradation we inhibited translation with cycloheximide (Obrig et al. 1971; Schneider‐Poetsch et al. 2010) for 1 h in HEK293 cells that had been transfected at 300 mosmol/kg either with scrambled siRNA or with siRNA against 14‐3‐3‐*β* and 14‐3‐3‐*ε* in combination. Then, we changed the medium, maintaining the cycloheximide, for 8 h to an otherwise identical one at 300 mosmol/kg or increased to 500 mosmol/kg by adding NaCl. The change in NFAT5 protein during 8 h under the various conditions is shown in Figure 3D. In the absence of cycloheximide, the siRNAs against 14‐3‐3‐*β* and 14‐3‐3‐*ε* in combination reduce the increase in NFAT5 protein at 500 mosmol/kg, in agreement with Figure 3A. In the absence of specific siRNAs, cycloheximide entirely prevents the high NaCl‐induced increase in NFAT5, confirming that the increase depends on translation. Also, when cycloheximide has stopped translation, the specific siRNAs do not reduce NFAT5 protein compared to scrambled control siRNA at 300 mosmol/kg, but they do at 500 mosmol/kg, which suggested that the 14‐3‐3s stabilize NFAT5 protein in the presence of high NaCl. We used cycloheximide chase to test this possibility directly (Fig. 3E and F). HEK293 cells were preincubated for 7 h at 300 or 500 mosmol/kg, then we inhibited translation with cycloheximide for 1 h. Cells maintained in cycloheximide were harvested 0–4 h later. In the presence of cycloheximide, siRNA‐mediated knockdown of 14‐3‐3‐*β* and 14‐3‐3‐*ε* causes time‐dependent decrease in NFAT5 protein at 500 mosmol/kg, compared to scrambled control siRNA. In contrast, at 300 mosmol/kg knockdown of 14‐3‐3‐*β* and 14‐3‐3‐*ε* does not accelerate the rate of fall of NFAT5 protein. We conclude that 14‐3‐3‐*β* and 14‐3‐3‐*ε* stabilize NFAT5 protein in the presence of high NaCl.

### Lack of coimmunoprecipitation of 14‐3‐3‐*β* or ‐*ε* with NFAT5

In order to test the possibility that the 14‐3‐3s might bind directly to NFAT5, we immunoprecipitated NFAT5‐V5 and identified associated proteins by immunoblot (Fig. 3G). We confirm our previous observation that CDK5 (Gallazzini et al. 2011) and HSP90 (Chen et al. 2007) coimmunopreciptiate with NFAT5. However, we do not observe coimmunoprecipitation of 14‐3‐3‐*β* or ‐*ε* with NFAT5‐V5, suggesting that the effect of the 14‐3‐3s on NFAT5 may be indirect via other proteins that signal activation of NFAT5, rather than a direct effect on NFAT5.

## Discussion

Having previously found that high NaCl causes rapid exit of 14‐3‐3 isoforms from the nucleus (Li et al. 2012), we used siRNA‐mediated knockdown to test the hypothesis that 14‐3‐3s might contribute to osmotic regulation of NFAT5. In support of the hypothesis we found that, when NaCl is elevated, knockdown of 14‐3‐3‐*β* and 14‐3‐3‐*ε* individually or in combination significantly decreases transcriptional activity of NFAT5, but knockdown of the other isoforms does not (Fig. 1B). Since siRNA knockdown of 14‐3‐3 isoforms was 62–77% (Fig. 1A) and therefore incomplete, we cannot eliminate possible effects of residual activity. That said, we searched for the mechanism(s) involved in 14‐3‐3‐*β* and ‐*ε* effects on NFAT5 transcriptional activity. Knockdown of 14‐3‐3‐*β* and/or ‐*ε* decreases NFAT5 transactivating activity while NaCl is elevated (Fig. 2A), indicating that 14‐3‐3‐*β* and ‐*ε* contribute to regulation of NFAT5 by affecting its transactivating activity. However, the combination of the siRNAs against ‐*β* and ‐*ε* inhibits NFAT5 transcriptional activity by 45% (Fig. 1B), but it only inhibits NFAT5 transactivating activity by 20% (Fig. 2A), which suggested that additional mechanisms might be involved. Knockdown of 14‐3‐3‐*β* and/or ‐*ε* does not affect the nuclear localization of NFAT5 (Fig. 2B and C), which excludes possible enhancement of nuclear translocation. However, knocking down 14‐3‐3‐*β* and/or ‐*ε* does reduce the protein abundance of NFAT5 (Fig. 3A and B) suggesting that 14‐3‐3‐*β* and ‐*ε* contribute to the high NaCl‐induced increase in NFAT5 protein. High NaCl increases NFAT5 mRNA, leading to increased translation (Miyakawa et al. 1999; Ko et al. 2000; Woo et al. 2000; Cai et al. 2005). However, knockdown of 14‐3‐3‐*β* and/or ‐*ε* does not affect NFAT5 mRNA (Fig. 3C), which suggested that the 14‐3‐3s might act, not by increasing translation of NFAT5, but by decreasing the rate at which it is degraded when NaCl is elevated. We confirmed that this is the case by cycloheximide chase (Fig. 3D–F). This high NaCl‐induced stabilization of NFAT5 protein was not detected previously using [^35^S]‐methionine pulse chase (Woo et al. 2000).

### Possibility of indirect, as well as direct, effects of 14‐3‐3‐*β* and ‐*ε* on NFAT5

14‐3‐3‐*β* and ‐*ε* could regulate NFAT5 directly by binding to phosphorylation sites contained in it and they could also bind to phosphorylation sites in signaling molecules that regulate NFAT5. Scansite (Obenauer et al. 2003) lists only one likely 14‐3‐3 binding site in NFAT5, namely T298. However, based on our previous study of this site (Izumi et al. 2012), the effects of 14‐3‐3‐*β* and ‐*ε* are unlikely to be mediated by binding to it. Thus, we did not find T298 to be phosphorylated, as is generally required for a direct action of 14‐3‐3s, and mutating this site reduces nuclear translocation of NFAT5, whereas knocking down 14‐3‐3‐*β* and/or ‐*ε* does not. Furthermore, we do not find that 14‐3‐3‐*β* or ‐*ε* coimmunoprecipitates with NFAT5 (Fig. 3G) which is also more consistent with an indirect effect of the 14‐3‐3s on NFAT5.

### 14‐3‐3s have been found to regulate signaling proteins involved in hypertonicity‐induced activation of NFAT5, but none of these signaling proteins regulates NFAT5 in the same manner as 14‐3‐3‐*β* and ‐*ε*

We researched the possibility that 14‐3‐3s might contribute indirectly to activation of NFAT5 by searching for references to effects of 14‐3‐3s on proteins that signal activation of NFAT5. Although we found that several proteins that signal activation of NFAT5 are regulated by 14‐3‐3s in one circumstance or another, none of the signaling proteins has the same effects on NFAT5 as 14‐3‐3‐*β* and ‐*ε*, that is, increase in NFAT5 protein abundance and transactivating activity, but not of its nuclear localization or mRNA abundance. Thus, MAPK1/ERK contributes to hypertonicity‐induced activation of NFAT5 in nucleus pulposus cells by increasing NFAT5 mRNA, protein, transactivating activity, and specific DNA binding (Tsai et al. 2007). Dominant negative 14‐3‐3*ζ* inhibits MAPK1/ERK in serum‐stimulated fibroblasts (Xing et al. 2000). PIK3R1/p85, the regulatory subunit of PI3K‐IA, contributes to hypertonicity‐induced activation of NFAT5 in Jurkat and HEK293 cells by increasing NFAT5 transactivating activity, but not its nuclear translocation or its protein abundance (Irarrazabal et al. 2006). 14‐3‐3*ζ* in cancer cells activates PIK3R1/p85 by binding to it (Neal et al. 2012). ABL1/c‐Abl contributes to hypertonicity‐induced activation of NFAT5 in HEK293 cells by increasing NFAT5 nuclear localization and transactivating activity (Gallazzini et al. 2010). ABL1/c‐Abl is usually retained in the cytoplasm by binding to 14‐3‐3 proteins on Thr‐735 (Yoshida et al. 2005). Activation of MAPK8/JNK, which occurs when NaCl is elevated (Zhang and Cohen 1996), induces phosphorylation of 14‐3‐3 proteins and their release from ABL1/c‐Abl. That promotes translocation of ABL1/c‐Abl to the nucleus, where it can activate transcription factors (Yoshida et al. 2005). MAP3K3/MEKK3 contributes to hypertonicity‐induced activation of NFAT5 in MDCK cells by increasing NFAT5 transcriptional activity via activation of MAPK14/p38 (Padda et al. 2006). MAPK14/p38 increases NFAT5 transactivating activity (Ko et al. 2002), mRNA and protein abundance (Tsai et al. 2007), and nuclear localization (Lee et al. 2008). 14‐3‐3*ε* inhibits dephosphorylation of MAP3K3/MEKK3‐S526, which maintains its catalytic activity (Fritz et al. 2006). RAC1 regulates NFAT5 activity through a RAC1‐OSM/PLC*γ*1 pathway. RAC1 increases NFAT5 transactivating activity (Zhou et al. 2011), protein (Zhou et al. 2011), and mRNA (Kino et al. 2009) abundance, but not nuclear localization (Zhou et al. 2011). RAC1 is regulated by 14‐3‐3‐*β* (Somanath and Byzova 2009) and 14‐3‐3‐*ζ* (O'Toole et al. 2011). We conclude that, although 14‐3‐3s can affect proteins that signal activation of NFAT5, no single one of these signaling proteins affects NFAT5 in the same way as 14‐3‐3‐*β* and ‐*ε*.

### Effects on 14‐3‐3s, themselves, of proteins that signal activation of NFAT5

Some of the protein kinases that regulate NFAT5, including AKT1 (Zhou et al. 2013) and PRKACA/PKA (Ferraris et al., 2002a; Zhou et al. 2013), can phosphorylate 14‐3‐3s on residues in their binding motifs (Aitken 2006). The phosphorylation prevents interaction of 14‐3‐3s with their targets. However, it is currently unclear whether high NaCl affects phosphorylation in the binding motifs within the 14‐3‐3s themselves.

### 14‐3‐3‐mediated stabilization of proteins

We find that 14‐3‐3‐*β* and/or ‐*ε* increases NFAT5 protein (Fig. 3A) by decreasing the rate at which it is degraded (Fig. 3D–F). Stabilization of proteins by 14‐3‐3s was previously observed for ATM‐phosphorylated E2F1 (Wang et al. 2004), PKA‐stimulated IRS2 (Neukamm et al. 2013), PKA‐stimulated CDKN1A/p27 (Short et al. 2010), and calcium/calmodulin‐dependent kinase‐stimulated HDAC7/histone deacetylase (Li et al. 2004). The 14‐3‐3s stabilize these proteins by inhibiting ubiquitination and proteosomal degradation. Similarly, 14‐3‐3σ binding blocks Mdm2‐dependent ubiquitination and nuclear export of p53 (Yang et al. 2003). It has been proposed that binding to 14‐3‐3s obscures the recognition motif for ubiquitin ligases in disordered regions of the proteins, thus protecting against proteasomal degradation (Neukamm et al. 2013). Disordered regions in transcription factors are important for their regulation (Bustos 2012), and NFAT5 contains large regions that are predicted to be disordered (disoPred2 http://bioinf.cs.ucl.ac.uk/psipred/?disopred=1; Pondr‐VL‐XT; http://www.pondr.com/index). Intrinsically unstructured regions are suitable for restructuring on interaction with a binding partner, and, since 14‐3‐3 proteins display a very rigid structure, they can act as anvils upon which a binding partner is restructured. 14‐3‐3s bind preferentially to disordered proteins (Bustos 2012).

### The isoform‐specific roles of 14‐3‐3‐*β* and ‐*ε*

When NaCl is elevated, siRNA‐mediated knockdown of 14‐3‐3‐*β* and/or ‐*ε* significantly reduces transcriptional activity of NFAT5, but knockdown of other 14‐3‐3 isoforms does not (Fig. 1B). The various 14‐3‐3 isoforms exhibit similar binding specificities, as would be anticipated since the residues lining their phosphopeptide‐binding grooves are highly conserved. Nevertheless, differences in the abilities of 14‐3‐3 isoforms to bind synthetic peptides and proteins have been reported, and other examples of isoform‐specific biologic responses are known (Bustos 2012). However, whether this is because of differences in binding specificity, subcellular localization, or another property is speculative (Tzivion and Avruch 2002). In addition, the propensity to heterodimerize seems to vary between isoforms. 14‐3‐3‐*σ* will only dimerize with itself, 14‐3‐3‐*ε* has a higher dimerization affinity toward other isoforms (*β*,* γ*,* τ*) compared with homodimerization, while the remaining isoforms have an equal binding affinity through hetero‐ and homodimerization (Yang et al. 2006). Although actions of 14‐3‐3 isoforms including 14‐3‐3‐*ε* (Lee et al. 2009) and 14‐3‐3‐*β* (Sugiyama et al. 2003, 2003), are previously reported, it is not clear that the actions are specific to those isoforms.

## Conflict of Interest

None declared.
